# Supplementary material for: Wide-ranging consequences of priority effects governed by an overarching factor
Source: eLife. 2022 Oct 27;11:e79647. doi: 10.7554/eLife.79647 (PMC9671501; doi:10.7554/eLife.79647)
Supplement: Figure 2—source data 1. [file elife-79647-fig2-data1.docx]

### Figure 2-source data 1 – Field sites in *Diplacus aurantiacus* field survey

| **Site number** | **Site name** | **Coordinates** | **Elevation (m)** | **Nectar sampling date** |
| --- | --- | --- | --- | --- |
| 1 | Bodega Bay (BB) | 38°20'11.00"N, 123° 2'48.15"W | 25 | July 08, 2015 |
| 2 | Muir Woods (MW) | 37°53'38.72"N, 122°33'50.54"W | 184 | June 30, 2015 |
| 3 | Sweeney Ridge (SR) | 37°36'51.33"N, 122°27'3.68"W | 216 | July 09, 2015 |
| 4 | Skyline Boulevard (SB) | 37°29'19.30"N, 122°21'49.38"W | 335 | July 16, 2015 |
| 5 | La Honda (LH) | 37°18'29.41"N, 122°16'22.21"W | 158 | June 23, 2015 |
| 6 | San Gregorio (SG) | 37°18'49.77"N, 122°23'7.67"W | 121 | June 24, 2015 |
| 7 | Soquel Valley (SV) | 37° 2'32.01"N, 121°57'56.45"W | 281 | July 01, 2015 |
| 8 | Swanton (SA) | 37° 5'0.42"N, 122°15'46.07"W | 136 | July 14, 2015 |
| 9 | Oak Hills (OH) | 36°46'46.64"N, 121°42'12.88"W | 48 | June 25, 2015 |
| 10 | Jacks Peak (JP) | 36°34'9.34"N, 121°51'34.77"W | 196 | July 02, 2015 |
| 11 | Carmel Highlands (CH) | 36°23'7.94"N, 121°54'2.48"W | 51 | July 07, 2015 |
| 12 | Big Sur (BS) | 36°19'53.73"N, 121°53'13.46"W | 106 | July 15, 2015 |
