# Supplementary material for: Wide-ranging consequences of priority effects governed by an overarching factor
Source: eLife. 2022 Oct 27;11:e79647. doi: 10.7554/eLife.79647 (PMC9671501; doi:10.7554/eLife.79647)
Supplement: Figure 2—source data 2. — To determine whether climatic factors and seasonality influence microbial abundance in this system, WorldClim bioclimatic variables (average annual mean temperature, temperature seasonality, and average monthly precipitation) were extracted for each plant and site. These variables, along with sampling date, were modeled as variables predicting bacterial and fungal abundance, respectively, in a linear mixed model with site as a random effect. [file elife-79647-fig2-data2.docx]

### Figure 2-source data 2 – Association between percentage of flowers colonized by yeast or bacteria per plant and the distance between host plants

To determine whether climatic factors and seasonality influence microbial abundance in this system, WorldClim bioclimatic variables (average annual mean temperature, temperature seasonality, and average monthly precipitation) were extracted for each plant and site. These variables, along with sampling date, were modeled as variables predicting bacterial and fungal abundance, respectively, in a linear mixed model with site as a random effect.

#### S1(a) Bacteria (Figure supplement 4A)

|  | **Estimate** | **Standard error** | **t value** | **p value** |
| --- | --- | --- | --- | --- |
| Intercept | 1.0322432 | 0.0146282 | 70.565 | <2e-16 |
| Difference in bacterial colonization ~ Geographic distance | 0.000268 | 0.0001467 | 1.827 | 0.0677 |

#### S1(b) Fungi (Figure supplement 4B)

|  | **Estimate** | **Standard error** | **t value** | **p value** |
| --- | --- | --- | --- | --- |
| Intercept | 0.4963776 | 0.0067623 | 73.404 | < 2e-16 |
| Difference in fungal colonization ~ Geographic distance | -0.0004053 | 0.0000678 | -5.978 | 2.36E-09 |
