# Supplementary material for: Wide-ranging consequences of priority effects governed by an overarching factor
Source: eLife. 2022 Oct 27;11:e79647. doi: 10.7554/eLife.79647 (PMC9671501; doi:10.7554/eLife.79647)
Supplement: Figure 2—source data 3. — Linear mixed model predicting bacterial or yeast abundance by average annual temperature (WorldClim bio1), temperature seasonality (WorldClim bio4), annual precipitation (WorldClim bio12), sampling date, with site location included as a random effect (n=144). [file elife-79647-fig2-data3.docx]

### Figure 2-source data 3 – Association between bioclimate variables, date of sampling, and microbial colonization

Linear mixed model predicting bacterial or yeast abundance by average annual temperature (WorldClim bio1), temperature seasonality (WorldClim bio4), annual precipitation (WorldClim bio12), sampling date, with site location included as a random effect (n=144).

#### S2(a) Bacteria:

|  | **Estimate** | **Standard error** | **t value** | **p value** |
| --- | --- | --- | --- | --- |
| Intercept | -5.89E+00 | 6.98E+00 | -0.843 | 0.399 |
| Date sampled | 2.14E-02 | 2.14E-02 | 1.001 | 0.317 |
| Average annual temperature (Bio1) | 2.64E-02 | 4.78E-02 | 0.552 | 0.581 |
| Temperature seasonality (Bio4) | -6.92E-05 | 4.39E-04 | -0.158 | 0.875 |
| Average monthly precipitation (Bio12) | 1.34E-03 | 1.14E-03 | 0.173 | 0.241 |

#### S2(b)Fungi:

|  | **Estimate** | **Standard error** | **t value** | **p value** |
| --- | --- | --- | --- | --- |
| Intercept | 3.92E-01 | 3.97E+00 | 0.099 | 0.921 |
| Date sampled | 1.13E-02 | 1.17E-02 | 0.967 | 0.333 |
| Average annual temperature (Bio1) | -1.57E-02 | 2.72E-02 | -0.576 | 0.564 |
| Temperature seasonality (Bio4) | 4.82E-05 | 2.41E-04 | 0.2 | 0.841 |
| Average monthly precipitation (Bio12) | 1.77E-04 | 5.97E-04 | 0.296 | 0.767 |
