# Supplementary material for: Wide-ranging consequences of priority effects governed by an overarching factor
Source: eLife. 2022 Oct 27;11:e79647. doi: 10.7554/eLife.79647 (PMC9671501; doi:10.7554/eLife.79647)
Supplement: Figure 3—source data 1. [file elife-79647-fig3-data1.docx]

### Figure 3-source data 1 – Primer sequences and PCR cycles for colony PCR

| **Target taxa** | **Primer Name** | **Sequence** |
| --- | --- | --- |
| Fungi | NL1 | GCA TAT CAA TAA GCG GAG GAA AAG |
| Fungi | NL4 | GGT CCG TGT TTC AAG ACG G |
| Bacteria | E1099FRC | GGG TTG CGC TCG TTR C |
| Bacteria | E343F | TAC GGR AGG CAG CAG |

**Yeast PCR cycles:**

| **# cycles** | **Temperature** | **Length (mins)** |
| --- | --- | --- |
| 1X | 95°C | 5:00 |
| 30X | 95°C | 0:45 |
| 30X | 50°C | 0:45 |
| 30X | 72°C | 0:45 |
| 1X | 72°C | 7:00 |

**Bacteria PCR cycles:**

| **# cycles** | **Temperature** | **Length (mins)** |
| --- | --- | --- |
| 1X | 95°C | 3:00 |
| 10X | 95°C | 0:30 |
| 10X | 56 to 51°C (decreasing -0.5°C per cycle) | 0:45 |
| 10X | 72°C | 0:45 |
| 20X | 95°C | 0:30 |
| 20X | 51°C | 0:45 |
| 20X | 72°C | 0:45 |
| 1X | 72°C | 7:00 |
