# Supplementary material for: Wide-ranging consequences of priority effects governed by an overarching factor
Source: eLife. 2022 Oct 27;11:e79647. doi: 10.7554/eLife.79647 (PMC9671501; doi:10.7554/eLife.79647)
Supplement: Figure 4—source data 1. — Treatments used in priority effects experiment with ancestral yeast, fully factorial experiment testing the effect of arrival order on priority effects. [file elife-79647-fig4-data1.docx]

### Figure 4-source data 1 - Priority effect experiment treatments

Treatments used in priority effects experiment with ancestral yeast, fully factorial experiment testing the effect of arrival order on priority effects.

| **Yeast** | | **Bacteria** | **Totals** | |  |
| --- | --- | --- | --- | --- | --- |
| **Strain** | **Arrival** | **Arrival** | **Total rounds** | **Biological replicates per round** | **Total biological replicates** |
| MR1 | First | Second | 5 | 2 | 10 |
| MR1 | Second | First | 5 | 2 | 10 |
| MR1 | First | First | 5 | 2 | 10 |
| MR1 | Second | Second | 5 | 2 | 10 |
| MR1 | First | None | 5 | 2 | 10 |
| MR1 | Second | None | 5 | 2 | 10 |
| MR1 | None | First | 5 | 2 | 10 |
| MR1 | None | Second | 5 | 2 | 10 |
| MY1082 | First | Second | 1 | 3 | 3 |
| MY1082 | Second | First | 1 | 3 | 3 |
| MY1082 | First | First | 1 | 3 | 3 |
| MY1082 | Second | Second | 1 | 3 | 3 |
| MY1082 | First | None | 1 | 3 | 3 |
| MY1082 | Second | None | 1 | 3 | 3 |
| MY1082 | None | First | 1 | 3 | 3 |
| MY1082 | None | Second | 1 | 3 | 3 |
| MY0202 | First | Second | 1 | 3 | 3 |
| MY0202 | Second | First | 1 | 3 | 3 |
| MY0202 | First | First | 1 | 3 | 3 |
| MY0202 | Second | Second | 1 | 3 | 3 |
| MY0202 | First | None | 1 | 3 | 3 |
| MY0202 | Second | None | 1 | 3 | 3 |
| MY0202 | None | First | 1 | 3 | 3 |
| MY0202 | None | Second | 1 | 3 | 3 |
