# Supplementary material for: Wide-ranging consequences of priority effects governed by an overarching factor
Source: eLife. 2022 Oct 27;11:e79647. doi: 10.7554/eLife.79647 (PMC9671501; doi:10.7554/eLife.79647)
Supplement: Figure 4—source data 2. — Results from a linear mixed model testing the effect of arrival order on yeast growth, where BY and YB represents initial arrival by bacteria or yeast, respectively. -Y and Y- represent the comparable growth of yeast at either arrival time (day 0 or day 2). Bold text shows p-values less than or equal to 0.05. [file elife-79647-fig4-data2.docx]

### Figure 4-source data 2 - Priority effect experiment results

Results from a linear mixed model testing the effect of arrival order on yeast growth, where BY and YB represents initial arrival by bacteria or yeast, respectively. -Y and Y- represent the comparable growth of yeast at either arrival time (day 0 or day 2). Bold text shows p-values less than or equal to 0.05.

#### S5(a) Yeast growth:

| **Treatment** | **Estimate** | **Standard error** | **Degrees of freedom** | **t ratio** | **p value** |
| --- | --- | --- | --- | --- | --- |
| YB – BY | 0.6213 | 0.0668 | 183 | 9.296 | **<.0001** |
| YB - (YB-) | 0.2429 | 0.0668 | 183 | 3.635 | **0.0048** |
| YB - (-YB) | 0.1195 | 0.0668 | 183 | 1.788 | 0.4763 |
| YB - (Y-) | 0.0352 | 0.0668 | 183 | 0.527 | 0.995 |
| YB - (-Y) | -0.2456 | 0.0668 | 183 | -3.674 | **0.0042** |
| BY - (YB-) | -0.3783 | 0.0668 | 183 | -5.661 | **<.0001** |
| BY - (-YB) | -0.5018 | 0.0668 | 183 | -7.508 | **<.0001** |
| BY - (Y-) | -0.586 | 0.0668 | 183 | -8.769 | **<.0001** |
| BY - (-Y) | -0.8668 | 0.0668 | 183 | -12.971 | **<.0001** |
| (YB-) - (-YB) | -0.1235 | 0.0668 | 183 | -1.848 | 0.438 |
| (YB-) - (Y-) | -0.2077 | 0.0668 | 183 | -3.108 | **0.0262** |
| (YB-) - (-Y) | -0.4885 | 0.0668 | 183 | -7.31 | **<.0001** |
| (-YB) - (Y-) | -0.0842 | 0.0668 | 183 | -1.26 | 0.806 |
| (-YB) - (-Y) | -0.365 | 0.0668 | 183 | -5.462 | **<.0001** |
| (Y-) - (-Y) | -0.2808 | 0.0668 | 183 | -4.202 | **0.0006** |

####

#### S5(b) Bacterial growth:

| **Treatment** | **Estimate** | **Standard error** | **Degrees of freedom** | **t ratio** | **p value** |
| --- | --- | --- | --- | --- | --- |
| YB – BY | -1.92858 | 0.0722 | 183 | -26.717 | <.0001 |
| YB - (YB-) | -1.40927 | 0.0722 | 183 | -19.523 | <.0001 |
| YB - (-YB) | -1.69876 | 0.0722 | 183 | -23.533 | <.0001 |
| YB - (B-) | -1.93511 | 0.0722 | 183 | -26.807 | <.0001 |
| YB - (-B) | -2.25898 | 0.0722 | 183 | -31.294 | <.0001 |
| BY - (YB-) | 0.51931 | 0.0722 | 183 | 7.194 | <.0001 |
| BY - (-YB) | 0.22982 | 0.0722 | 183 | 3.184 | 0.0209 |
| BY - (B-) | -0.00652 | 0.0722 | 183 | -0.09 | 1 |
| BY - (-B) | -0.3304 | 0.0722 | 183 | -4.577 | 0.0001 |
| (YB-) - (-YB) | -0.28949 | 0.0722 | 183 | -4.01 | 0.0012 |
| (YB-) - (B-) | -0.52583 | 0.0722 | 183 | -7.284 | <.0001 |
| (YB-) - (-B) | -0.84971 | 0.0722 | 183 | -11.771 | <.0001 |
| (-YB) - (B-) | -0.23634 | 0.0722 | 183 | -3.274 | 0.0158 |
| (-YB) - (-B) | -0.56022 | 0.0722 | 183 | -7.761 | <.0001 |
| (B-) - (-B) | -0.32388 | 0.0722 | 183 | -4.487 | 0.0002 |

#### 
