# Supplementary material for: Wide-ranging consequences of priority effects governed by an overarching factor
Source: eLife. 2022 Oct 27;11:e79647. doi: 10.7554/eLife.79647 (PMC9671501; doi:10.7554/eLife.79647)
Supplement: Figure 6—source data 1. — Results from a linear mixed model testing the effect of yeast strain (MR1, MY0182, MY0202) on the strength of priority effects exerted by A. nectaris calculated using this metric:,PE=log(BY-Y)-log(YBY-) where BY and YB represents initial dominance by bacteria or yeast, respectively. -Y and Y- represent the comparable growth of yeast at either density, alone and treatment densities were averaged by round of the experiment. Bold text shows p-values less than or equal to 0.05. [file elife-79647-fig6-data1.docx]

### Figure 6-source data 1 - Priority effect experiment results with wild strains

Results from a linear mixed model testing the effect of yeast strain (MR1, MY0182, MY0202) on the strength of priority effects exerted by *A. nectaris* calculated using this metric: $PE = log(\frac{\mathrm{BY}}{-Y}) - log(\frac{\mathrm{YB}}{Y-})$, where BY and YB represents initial dominance by bacteria or yeast, respectively. -Y and Y- represent the comparable growth of yeast at either density, alone and treatment densities were averaged by round of the experiment. Bold text shows p-values less than or equal to 0.05.

| **Comparison** | **Estimate** | **Standard error** | **Degrees of freedom** | **t ratio** | **p value** |
| --- | --- | --- | --- | --- | --- |
| MR1 - MY0182 | -0.453 | 0.0458 | 19 | -9.901 | **<.0001** |
| MR1 - MY0202 | -0.533 | 0.0473 | 19 | -11.285 | **<.0001** |
| MY0182 - MY0202 | -0.08 | 0.0457 | 19 | -1.752 | 0.2126 |
