# Supplementary material for: Wide-ranging consequences of priority effects governed by an overarching factor
Source: eLife. 2022 Oct 27;11:e79647. doi: 10.7554/eLife.79647 (PMC9671501; doi:10.7554/eLife.79647)
Supplement: Figure 7—source data 1. — Treatments used in priority effects experiment with experimentally-evolved yeast, fully factorial experiment testing the effect of initial density (10,000 cells/µL (‘early’) or 10 cells/µL (‘late’)), evolution treatment (ancestral, evolved in normal nectar, low-pH nectar, or bacteria-conditioned nectar), and evolutionary replicate (independent evolutionary lineages) on priority effects. [file elife-79647-fig7-data1.docx]

### Figure 7-source data 1 - Evolution treatments

Treatments used in priority effects experiment with experimentally-evolved yeast, fully factorial experiment testing the effect of initial density (10,000 cells/µL (“early”) or 10 cells/µL (“late”)), evolution treatment (ancestral, evolved in normal nectar, low-pH nectar, or bacteria-conditioned nectar), and evolutionary replicate (independent evolutionary lineages) on priority effects.

| **Yeast** | | | | **Bacteria** | | | **Totals** | |
| --- | --- | --- | --- | --- | --- | --- | --- | --- |
| **Species** | **Evolution** | **Evolutionary Replicate** | **Density** | **Species** | **Evolution** | **Density** | **Biological replicates per round** | **Total biological replicates** |
| *M. reukaufii* | ancestral | none | high | *A. nectaris* | ancestral | low | 9 | 36 |
| *M. reukaufii* | normal | 1 | high | *A. nectaris* | ancestral | low | 2 | 8 |
| *M. reukaufii* | low pH | 1 | high | *A. nectaris* | ancestral | low | 2 | 8 |
| *M. reukaufii* | conditioned | 1 | high | *A. nectaris* | ancestral | low | 2 | 8 |
| *M. reukaufii* | ancestral | none | high | *A. nectaris* | ancestral | low | 9 | 36 |
| *M. reukaufii* | normal | 2 | high | *A. nectaris* | ancestral | low | 2 | 8 |
| *M. reukaufii* | low pH | 2 | high | *A. nectaris* | ancestral | low | 2 | 8 |
| *M. reukaufii* | conditioned | 2 | high | *A. nectaris* | ancestral | low | 2 | 8 |
| *M. reukaufii* | ancestral | none | high | *A. nectaris* | ancestral | low | 9 | 36 |
| *M. reukaufii* | normal | 3 | high | *A. nectaris* | ancestral | low | 2 | 8 |
| *M. reukaufii* | low pH | 3 | high | *A. nectaris* | ancestral | low | 2 | 8 |
| *M. reukaufii* | conditioned | 3 | high | *A. nectaris* | ancestral | low | 2 | 8 |
| *M. reukaufii* | ancestral | none | high | *A. nectaris* | ancestral | low | 9 | 36 |
| *M. reukaufii* | normal | 4 | high | *A. nectaris* | ancestral | low | 2 | 8 |
| *M. reukaufii* | low pH | 4 | high | *A. nectaris* | ancestral | low | 2 | 8 |
| *M. reukaufii* | conditioned | 4 | high | *A. nectaris* | ancestral | low | 2 | 8 |
| *M. reukaufii* | ancestral | none | high | *A. nectaris* | ancestral | low | 9 | 36 |
| *M. reukaufii* | normal | 1 | high | *A. nectaris* | ancestral | low | 2 | 8 |
| *M. reukaufii* | low pH | 1 | high | *A. nectaris* | ancestral | low | 2 | 8 |
| *M. reukaufii* | conditioned | 1 | high | *A. nectaris* | ancestral | low | 2 | 8 |
| *M. reukaufii* | ancestral | none | high | *A. nectaris* | ancestral | low | 9 | 36 |
| *M. reukaufii* | normal | 2 | high | *A. nectaris* | ancestral | low | 2 | 8 |
| *M. reukaufii* | low pH | 2 | high | *A. nectaris* | ancestral | low | 2 | 8 |
| *M. reukaufii* | conditioned | 2 | high | *A. nectaris* | ancestral | low | 2 | 8 |
| *M. reukaufii* | ancestral | none | high | *A. nectaris* | ancestral | low | 9 | 36 |
| *M. reukaufii* | normal | 3 | high | *A. nectaris* | ancestral | low | 2 | 8 |
| *M. reukaufii* | low pH | 3 | high | *A. nectaris* | ancestral | low | 2 | 8 |
| *M. reukaufii* | conditioned | 3 | high | *A. nectaris* | ancestral | low | 2 | 8 |
| *M. reukaufii* | ancestral | none | high | *A. nectaris* | ancestral | low | 9 | 36 |
| *M. reukaufii* | normal | 4 | high | *A. nectaris* | ancestral | low | 2 | 8 |
| *M. reukaufii* | low pH | 4 | high | *A. nectaris* | ancestral | low | 2 | 8 |
| *M. reukaufii* | conditioned | 4 | high | *A. nectaris* | ancestral | low | 2 | 8 |
| *M. reukaufii* | ancestral | none | low | *A. nectaris* | ancestral | high | 9 | 36 |
| *M. reukaufii* | normal | 1 | low | *A. nectaris* | ancestral | high | 2 | 8 |
| *M. reukaufii* | low pH | 1 | low | *A. nectaris* | ancestral | high | 2 | 8 |
| *M. reukaufii* | conditioned | 1 | low | *A. nectaris* | ancestral | high | 2 | 8 |
| *M. reukaufii* | ancestral | none | low | *A. nectaris* | ancestral | high | 9 | 36 |
| *M. reukaufii* | normal | 2 | low | *A. nectaris* | ancestral | high | 2 | 8 |
| *M. reukaufii* | low pH | 2 | low | *A. nectaris* | ancestral | high | 2 | 8 |
| *M. reukaufii* | conditioned | 2 | low | *A. nectaris* | ancestral | high | 2 | 8 |
| *M. reukaufii* | ancestral | none | low | *A. nectaris* | ancestral | high | 9 | 36 |
| *M. reukaufii* | normal | 3 | low | *A. nectaris* | ancestral | high | 2 | 8 |
| *M. reukaufii* | low pH | 3 | low | *A. nectaris* | ancestral | high | 2 | 8 |
| *M. reukaufii* | conditioned | 3 | low | *A. nectaris* | ancestral | high | 2 | 8 |
| *M. reukaufii* | ancestral | none | low | *A. nectaris* | ancestral | high | 9 | 36 |
| *M. reukaufii* | normal | 4 | low | *A. nectaris* | ancestral | high | 2 | 8 |
| *M. reukaufii* | low pH | 4 | low | *A. nectaris* | ancestral | high | 2 | 8 |
| *M. reukaufii* | conditioned | 4 | low | *A. nectaris* | ancestral | high | 2 | 8 |
| *M. reukaufii* | ancestral | none | low | *A. nectaris* | ancestral | high | 9 | 36 |
| *M. reukaufii* | normal | 1 | low | *A. nectaris* | ancestral | high | 2 | 8 |
| *M. reukaufii* | low pH | 1 | low | *A. nectaris* | ancestral | high | 2 | 8 |
| *M. reukaufii* | conditioned | 1 | low | *A. nectaris* | ancestral | high | 2 | 8 |
| *M. reukaufii* | ancestral |  | low | *A. nectaris* | ancestral | high | 9 | 36 |
| *M. reukaufii* | ancestral | none | low | *A. nectaris* | ancestral | high | 2 | 8 |
| *M. reukaufii* | normal | 2 | low | *A. nectaris* | ancestral | high | 2 | 8 |
| *M. reukaufii* | low pH | 2 | low | *A. nectaris* | ancestral | high | 2 | 8 |
| *M. reukaufii* | conditioned | 2 | low | *A. nectaris* | ancestral | high | 9 | 36 |
| *M. reukaufii* | ancestral | none | low | *A. nectaris* | ancestral | high | 2 | 8 |
| *M. reukaufii* | normal | 3 | low | *A. nectaris* | ancestral | high | 2 | 8 |
| *M. reukaufii* | low pH | 3 | low | *A. nectaris* | ancestral | high | 2 | 8 |
| *M. reukaufii* | conditioned | 3 | low | *A. nectaris* | ancestral | high | 9 | 36 |
| *M. reukaufii* | ancestral | none | low | *A. nectaris* | ancestral | high | 2 | 8 |
| *M. reukaufii* | normal | 4 | low | *A. nectaris* | ancestral | high | 2 | 8 |
| *M. reukaufii* | low pH | 4 | low | *A. nectaris* | ancestral | high | 2 | 8 |
| *M. reukaufii* | conditioned | 4 | low | *A. nectaris* | ancestral | high | 2 | 8 |
| *M. reukaufii* | ancestral | none | high | nectar | nectar | nectar | 6 | 24 |
| *M. reukaufii* | normal | 1 | high | nectar | nectar | nectar | 2 | 8 |
| *M. reukaufii* | low pH | 1 | high | nectar | nectar | nectar | 2 | 8 |
| *M. reukaufii* | conditioned | 1 | high | nectar | nectar | nectar | 2 | 8 |
| *M. reukaufii* | ancestral | none | high | nectar | nectar | nectar | 6 | 24 |
| *M. reukaufii* | normal | 2 | high | nectar | nectar | nectar | 2 | 8 |
| *M. reukaufii* | low pH | 2 | high | nectar | nectar | nectar | 2 | 8 |
| *M. reukaufii* | conditioned | 2 | high | nectar | nectar | nectar | 2 | 8 |
| *M. reukaufii* | ancestral | none | high | nectar | nectar | nectar | 6 | 24 |
| *M. reukaufii* | normal | 3 | high | nectar | nectar | nectar | 2 | 8 |
| *M. reukaufii* | low pH | 3 | high | nectar | nectar | nectar | 2 | 8 |
| *M. reukaufii* | conditioned | 3 | high | nectar | nectar | nectar | 2 | 8 |
| *M. reukaufii* | ancestral | none | high | nectar | nectar | nectar | 6 | 24 |
| *M. reukaufii* | normal | 4 | high | nectar | nectar | nectar | 2 | 8 |
| *M. reukaufii* | low pH | 4 | high | nectar | nectar | nectar | 2 | 8 |
| *M. reukaufii* | conditioned | 4 | high | nectar | nectar | nectar | 2 | 8 |
| *M. reukaufii* | ancestral | none | low | nectar | nectar | nectar | 6 | 24 |
| *M. reukaufii* | normal | 1 | low | nectar | nectar | nectar | 2 | 8 |
| *M. reukaufii* | low pH | 1 | low | nectar | nectar | nectar | 2 | 8 |
| *M. reukaufii* | conditioned | 1 | low | nectar | nectar | nectar | 2 | 8 |
| *M. reukaufii* | ancestral | none | low | nectar | nectar | nectar | 6 | 24 |
| *M. reukaufii* | normal | 2 | low | nectar | nectar | nectar | 2 | 8 |
| *M. reukaufii* | low pH | 2 | low | nectar | nectar | nectar | 2 | 8 |
| *M. reukaufii* | conditioned | 2 | low | nectar | nectar | nectar | 2 | 8 |
| *M. reukaufii* | ancestral | none | low | nectar | nectar | nectar | 6 | 24 |
| *M. reukaufii* | normal | 3 | low | nectar | nectar | nectar | 2 | 8 |
| *M. reukaufii* | low pH | 3 | low | nectar | nectar | nectar | 2 | 8 |
| *M. reukaufii* | conditioned | 3 | low | nectar | nectar | nectar | 2 | 8 |
| *M. reukaufii* | ancestral | none | low | nectar | nectar | nectar | 6 | 24 |
| *M. reukaufii* | normal | 4 | low | nectar | nectar | nectar | 2 | 8 |
| *M. reukaufii* | low pH | 4 | low | nectar | nectar | nectar | 2 | 8 |
| *M. reukaufii* | conditioned | 4 | low | nectar | nectar | nectar | 2 | 8 |
| Nectar | nectar | nectar | nectar | *A. nectaris* | ancestral | high | 6 | 24 |
| Nectar | nectar | nectar | nectar | *A. nectaris* | ancestral | low | 6 | 24 |
