# Supplementary material for: Wide-ranging consequences of priority effects governed by an overarching factor
Source: eLife. 2022 Oct 27;11:e79647. doi: 10.7554/eLife.79647 (PMC9671501; doi:10.7554/eLife.79647)
Supplement: Figure 7—source data 2. — Results from a linear mixed model testing the effect of evolution treatment (ancestral, evolved in normal nectar, low-pH nectar, or bacteria-conditioned nectar) on the strength of priority effects calculated using one of two metrics: Priority effect metric #1: (Figure 7),PE1=log(BY-Y)-log(YBY-) where BY and YB represent initial dominance by bacteria or yeast, respectively. -Y and Y- represent the comparable growth of yeast at either density, alone and treatment densities were averaged by round of the experiment. Bold text shows p-values less than or equal to 0.05. Priority effect metric #2: (Figure 7—figure supplement 1).,PE2=log(BYYB) where BY and YB represent initial dominance by bacteria or yeast, respectively. Treatment densities were averaged by round of the experiment. Bold text shows p-values less than or equal to 0.05. [file elife-79647-fig7-data2.docx]

### Figure 7-source data 2 - Priority effect experiment results with evolved strains (priority effect metric)

Results from a linear mixed model testing the effect of evolution treatment (ancestral, evolved in normal nectar, low-pH nectar, or bacteria-conditioned nectar) on the strength of priority effects calculated using one of two metrics:

#### Priority effect metric #1: (Figure 7)

$PE1 = log(\frac{\mathrm{BY}}{-Y}) - log(\frac{\mathrm{YB}}{Y-})$, where BY and YB represent initial dominance by bacteria or yeast, respectively. -Y and Y- represent the comparable growth of yeast at either density, alone and treatment densities were averaged by round of the experiment. Bold text shows p-values less than or equal to 0.05.

| **Comparison** | **Estimate** | **Standard error** | **Degrees of freedom** | **t ratio** | **p value** |
| --- | --- | --- | --- | --- | --- |
| ancestral - normal | -0.0477 | 0.0679 | 65 | -0.703 | 0.8955 |
| ancestral - low pH | -0.3132 | 0.0679 | 65 | -4.614 | **0.0001** |
| ancestral - bacteria conditioned | -0.1739 | 0.0679 | 65 | -2.562 | 0.0599 |
| normal - low pH | -0.2655 | 0.0744 | 65 | -3.57 | **0.0037** |
| normal - bacteria conditioned | -0.1262 | 0.0744 | 65 | -1.697 | 0.3336 |
| low pH - bacteria conditioned | 0.1393 | 0.0744 | 65 | 1.873 | 0.2495 |

#### Priority effect metric #2: (Figure 7-figure supplement 1)

$PE2 = log(\frac{\mathrm{BY}}{\mathrm{YB}})$, where BY and YB represent initial dominance by bacteria or yeast, respectively. Treatment densities were averaged by round of the experiment. Bold text shows p-values less than or equal to 0.05.

| **Comparison** | **Estimate** | **Standard error** | **Degrees of freedom** | **t ratio** | **p value** |
| --- | --- | --- | --- | --- | --- |
| ancestral - normal | -0.11069 | 0.0614 | 77 | -1.803 | 0.28 |
| ancestral - low_pH | -0.18418 | 0.0614 | 77 | -2.999 | **0.0187** |
| ancestral - AN_con | -0.17655 | 0.0614 | 77 | -2.875 | **0.0263** |
| normal - low_pH | -0.07349 | 0.0723 | 77 | -1.017 | 0.74 |
| normal - AN_con | -0.06586 | 0.0723 | 77 | -0.911 | 0.7988 |
| low_pH - AN_con | 0.00762 | 0.0723 | 77 | 0.106 | 0.9996 |
