# Supplementary material for: Wide-ranging consequences of priority effects governed by an overarching factor
Source: eLife. 2022 Oct 27;11:e79647. doi: 10.7554/eLife.79647 (PMC9671501; doi:10.7554/eLife.79647)
Supplement: Figure 7—source data 3. — Results from a linear mixed model testing the effect of yeast initial density (10,000 colony forming units/µL (‘early’) or 10 cells/µL (‘late’), yeast monoculture or competition with bacteria) treatment and evolution treatment (ancestral, evolved in normal nectar, low-pH nectar, or bacteria-conditioned nectar) on the difference in final yeast density between treatments with a high density of bacteria and a low density of yeast (BY) and yeast grown in monoculture at a low density (-Y). Growth difference was calculated as (BY) - (-Y). Bold text shows p-values less than or equal to 0.05. [file elife-79647-fig7-data3.docx]

### Figure 7-source data 3 - Differences in growth between evolved strains with and without bacteria

Results from a linear mixed model testing the effect of yeast initial density (10,000 colony forming units/µL (“early”) or 10 cells/µL (“late”), yeast monoculture or competition with bacteria) treatment and evolution treatment (ancestral, evolved in normal nectar, low-pH nectar, or bacteria-conditioned nectar) on the difference in final yeast density between treatments with a high density of bacteria and a low density of yeast (BY) and yeast grown in monoculture at a low density (-Y). Growth difference was calculated as (BY) - (-Y). Bold text shows p-values less than or equal to 0.05.

| **Comparison** | **Estimate** | **Standard error** | **Degrees of freedom** | **t ratio** | **p value** |
| --- | --- | --- | --- | --- | --- |
| ancestral - normal | -0.0303 | 0.0969 | 127 | -0.313 | 0.9894 |
| ancestral - low_pH | -0.4636 | 0.1021 | 127 | -4.539 | **0.0001** |
| ancestral - AN_con | -0.3166 | 0.0969 | 127 | -3.267 | **0.0076** |
| normal - low_pH | -0.4333 | 0.1099 | 127 | -3.943 | **0.0008** |
| normal - AN_con | -0.2863 | 0.104 | 127 | -2.754 | **0.0337** |
| low_pH - AN_con | 0.147 | 0.1099 | 127 | 1.338 | 0.5409 |

### 
