# Supplementary material for: Wide-ranging consequences of priority effects governed by an overarching factor
Source: eLife. 2022 Oct 27;11:e79647. doi: 10.7554/eLife.79647 (PMC9671501; doi:10.7554/eLife.79647)
Supplement: Figure 7—source data 4. — Results from a linear mixed model testing the effect of yeast initial density (10,000 colony forming units/µL (‘high’) or 10 cells/µL (‘low’), yeast monoculture or competition with bacteria) treatment and evolution treatment (ancestral, evolved in normal nectar, low-pH nectar, or bacteria-conditioned nectar) on final density of yeast. Bold text shows p-values less than or equal to 0.05. [file elife-79647-fig7-data4.docx]

### Figure 7-source data 4 - Difference in final yeast densities between evolved strains with and without bacteria

Results from a linear mixed model testing the effect of yeast initial density (10,000 colony forming units/µL (“high”) or 10 cells/µL (“low”), yeast monoculture or competition with bacteria) treatment and evolution treatment (ancestral, evolved in normal nectar, low-pH nectar, or bacteria-conditioned nectar) on final density of yeast. Bold text shows p-values less than or equal to 0.05.

| **Treatment** | **Comparison** | **Estimate** | **Standard error** | **Degrees of freedom** | **t ratio** | **p value** |
| --- | --- | --- | --- | --- | --- | --- |
| **-Y** | bacteria conditioned - ancestral | 0.1204 | 0.0967 | 593 | 1.244 | 0.5989 |
|  | bacteria conditioned - low pH | 0.2535 | 0.1055 | 593 | 2.402 | 0.0778 |
|  | bacteria conditioned - normal | -0.1001 | 0.1055 | 593 | -0.949 | 0.7785 |
|  | ancestral - low pH | 0.1331 | 0.0967 | 593 | 1.376 | 0.515 |
|  | ancestral - normal | -0.2205 | 0.0967 | 593 | -2.279 | 0.1041 |
|  | low pH - normal | -0.3536 | 0.1055 | 593 | -3.351 | **0.0047** |
| **BY** | bacteria conditioned - ancestral | 0.4175 | 0.0903 | 593 | 4.624 | **<.0001** |
|  | bacteria conditioned - low pH | 0.0967 | 0.1105 | 593 | 0.875 | 0.8176 |
|  | bacteria conditioned - normal | 0.1862 | 0.1055 | 593 | 1.765 | 0.2916 |
|  | ancestral - low pH | -0.3207 | 0.0959 | 593 | -3.344 | **0.0049** |
|  | ancestral - normal | -0.2313 | 0.0903 | 593 | -2.562 | 0.0519 |
|  | low pH - normal | 0.0895 | 0.1105 | 593 | 0.81 | 0.8499 |
| **Y-** | bacteria conditioned - ancestral | 0.1658 | 0.0967 | 593 | 1.714 | 0.3173 |
|  | bacteria conditioned - low pH | -0.0013 | 0.1055 | 593 | -0.012 | 1 |
|  | bacteria conditioned - normal | 0.07 | 0.1064 | 593 | 0.658 | 0.9127 |
|  | ancestral - low pH | -0.1671 | 0.0967 | 593 | -1.727 | 0.3104 |
|  | ancestral - normal | -0.0958 | 0.0977 | 593 | -0.981 | 0.7605 |
|  | low pH - normal | 0.0713 | 0.1064 | 593 | 0.67 | 0.9083 |
| **YB** | bacteria conditioned - ancestral | 0.5153 | 0.0897 | 593 | 5.747 | **<.0001** |
|  | bacteria conditioned - low pH | 0.3152 | 0.1055 | 593 | 2.987 | **0.0155** |
|  | bacteria conditioned - normal | 0.1816 | 0.1055 | 593 | 1.721 | 0.3134 |
|  | ancestral - low pH | -0.2001 | 0.0897 | 593 | -2.231 | 0.116 |
|  | ancestral - normal | -0.3337 | 0.0897 | 593 | -3.721 | **0.0012** |
|  | low pH - normal | -0.1336 | 0.1055 | 593 | -1.266 | 0.585 |

### 
