# Supplementary material for: Wide-ranging consequences of priority effects governed by an overarching factor
Source: eLife. 2022 Oct 27;11:e79647. doi: 10.7554/eLife.79647 (PMC9671501; doi:10.7554/eLife.79647)
Supplement: Figure 8—source data 1. — The relationship between monoculture growth and resistance to priority effects was calculated using a linear mixed model predicting the difference in the strength of priority effects from the monoculture growth. Both of these values were comparing each evolved strain to the ancestral strain within a single weekly iteration of the experiment, which was also included as a random effect. Additional model associated with Figure 8—figure supplement 1: (raw data for evolved strains not adjusted for ancestral). [file elife-79647-fig8-data1.docx]

### Figure 8-source data 1 - Relationship between resistance to priority effects and monoculture growth

The relationship between monoculture growth and resistance to priority effects was calculated using a linear mixed model predicting the difference in the strength of priority effects from the monoculture growth. Both of these values were comparing each evolved strain to the ancestral strain within a single weekly iteration of the experiment, which was also included as a random effect.

|  | **Estimate** | **Standard error** | **z value** | **p value** |
| --- | --- | --- | --- | --- |
| Intercept | 0.18475 | 0.0263 | 7.024 | 2.16E-12 |
| Difference in priority effect susceptibility ~ Difference in intrinsic growth | -0.10204 | 0.05278 | -1.933 | 0.0532 |

**Additional model associated with Figure 8-figure supplement 1:** (raw data for evolved strains not adjusted for ancestral)

|  | **Estimate** | **Standard error** | **z value** | **p value** |
| --- | --- | --- | --- | --- |
| Intercept | 0.10454 | 0.21986 | 0.476 | 6.34E-01 |
| Difference in priority effect suseptibility ~ Difference in intrinsic growth | -0.21525 | 0.07781 | -2.766 | 0.00567 |
