# Supplementary material for: Wide-ranging consequences of priority effects governed by an overarching factor
Source: eLife. 2022 Oct 27;11:e79647. doi: 10.7554/eLife.79647 (PMC9671501; doi:10.7554/eLife.79647)
Supplement: Figure 9—source data 1. — Mapping summary of evolved and ancestral genomes. Link to full MultiQC report. The average per sample, genome-wide coverage was 444X. [file elife-79647-fig9-data1.docx]

### Figure 9-source data 1 - Sequencing and mapping QC

Mapping summary of evolved and ancestral genomes. [Link](https://drive.google.com/file/d/1RftIbyph1yBWrNV5xul38FvX0cztztQ_/view?usp=sharing) to full MultiQC report. The average per sample, genome-wide coverage was 444X.

| **Treatment** | **Evolutionary replicate** | **M Reads Mapped** | **≥ 30X** | **Median cov** | **Mean cov** | **% Aligned** | **% Dups** | **Error rate** |
| --- | --- | --- | --- | --- | --- | --- | --- | --- |
| low pH | 1 | 48 | 99.40% | 396.0X | 406.1X | 98.80% | 13.80% | 0.47% |
| bacteria conditioned | 2 | 55.4 | 99.40% | 475.0X | 479.8X | 98.70% | 15.00% | 0.48% |
| bacteria conditioned | 3 | 60.6 | 99.40% | 517.0X | 523.4X | 98.90% | 16.80% | 0.48% |
| bacteria conditioned | 4 | 51.6 | 99.40% | 428.0X | 438.4X | 98.30% | 19.90% | 0.48% |
| Ancestral | NA | 51.9 | 99.30% | 449.0X | 450.7X | 98.70% | 13.90% | 0.48% |
| low pH | 2 | 54.3 | 99.40% | 457.0X | 465.0X | 98.80% | 15.20% | 0.48% |
| low pH | 3 | 51.1 | 99.40% | 425.0X | 433.4X | 98.90% | 14.80% | 0.47% |
| low pH | 4 | 52.4 | 99.40% | 432.0X | 444.2X | 98.80% | 15.70% | 0.48% |
| Normal | 1 | 56.8 | 99.40% | 474.0X | 483.4X | 98.80% | 15.80% | 0.48% |
| Normal | 2 | 51.8 | 99.40% | 430.0X | 439.7X | 98.70% | 14.70% | 0.48% |
| Normal | 3 | 46.1 | 99.30% | 385.0X | 393.4X | 98.80% | 14.00% | 0.48% |
| Normal | 4 | 47.4 | 99.30% | 395.0X | 401.5X | 98.70% | 14.90% | 0.48% |
| bacteria conditioned | 1 | 47.2 | 99.30% | 398.0X | 406.8X | 98.70% | 14.60% | 0.49% |

### 
