# Supplementary material for: Wide-ranging consequences of priority effects governed by an overarching factor
Source: eLife. 2022 Oct 27;11:e79647. doi: 10.7554/eLife.79647 (PMC9671501; doi:10.7554/eLife.79647)
Supplement: Figure 9—source data 2. — Nearest annotated gene to each loci with treatment specific divergence (loss of heterozygosity permutation test) with P<0.1 and FST = 0.3–0.5. [file elife-79647-fig9-data2.docx]

### Figure 9-source data 2 - Nearest annotated gene with treatment-specific divergence

​​Nearest annotated gene to each loci with treatment specific divergence (loss of heterozygosity permutation test) with p<0.1 and F_ST_ = 0.3-0.5.

| **Scaffold** | **SNP** | **Treatment comparison** | **F_ST_** | **LOH p-value** | **InterPro Description** | **Loci distance to gene (bp)** |
| --- | --- | --- | --- | --- | --- | --- |
| 2 | 192248 | low pH vs. bacteria-conditioned | 0.5 | NA | NA | 2131 |
| 2 | 721157 | low pH vs. bacteria-conditioned | 0.333333 | NA | Ribosomal protein L7Ae/L30e/S12e/Gadd45 | 241 |
| 2 | 977423 | low pH vs. bacteria-conditioned | 0.25 | 0.0969031 | Alpha/beta hydrolase fold-1 | 4633 |
| 2 | 1335729 | normal vs. low pH | 0.333333 | 0.06193806 | SH3 domain | 4956 |
| 2 | 1458114 | normal vs. low pH | 0.25 | 0.07992008 | Ribosomal protein S9 | 441 |
| 2 | 1484768 | low pH vs. bacteria-conditioned | 0.25 | 0.06993007 | Leucine-rich repeat, cysteine-containing subtype | 1502 |
| 2 | 1610283 | low pH vs. bacteria-conditioned | 0.25 | 0.02897103 | Chorismate mutase, AroQ class, eukaryotic type | 4335 |
| 2 | 1610283 | normal vs. low pH | 0.25 | 0.02897103 | Chorismate mutase, AroQ class, eukaryotic type | 4335 |
| 2 | 1613656 | normal vs. bacteria-conditioned | 0.333333 | NA | Phox homologous domain | 3370 |
| 2 | 1613656 | normal vs. low pH | 0.333333 | NA | Phox homologous domain | 3370 |
| 2 | 2103199 | low pH vs. bacteria-conditioned | 0.25 | 0.06993007 | HAT (Half-A-TPR) repeat | 4950 |
| 2 | 2378124 | normal vs. bacteria-conditioned | 0.25 | 0.06193806 | NOB1 zinc finger-like superfamily | 3480 |
| 3 | 17088 | normal vs. bacteria-conditioned | 0.5 | NA | Tubulin/FtsZ, GTPase domain superfamily | 2796 |
| 3 | 599816 | normal vs. low pH | 0.333333 | NA | HPP | 3373 |
| 3 | 2013434 | normal vs. bacteria-conditioned | 0.333333 | NA | NADP-dependent oxidoreductase domain superfamily | 3350 |
| 3 | 2621204 | normal vs. low pH | 0.333333 | NA | TRP, C-terminal | 4145 |
| 3 | 2957032 | low pH vs. bacteria-conditioned | 0.333333 | NA | Romo1/Mgr2 | 4081 |
| 4 | 16918 | normal vs. low pH | 0.25 | 0.08991009 | SUN domain | 1281 |
| 4 | 26373 | low pH vs. bacteria-conditioned | 0.333333 | NA | NA | 4551 |
| 4 | 124928 | normal vs. bacteria-conditioned | 0.333333 | NA | MIF4G-like, type 3 | 2444 |
| 4 | 376020 | normal vs. low pH | 0.333333 | NA | ATP synthase, F1 complex, epsilon subunit superfamily, mitochondrial | 2960 |
| 4 | 471834 | normal vs. low pH | 0.25 | 0.06193806 | Major facilitator superfamily domain | 2272 |
| 4 | 552417 | low pH vs. bacteria-conditioned | 0.333333 | NA | Hap4 transcription factor, heteromerization domain | 200 |
| 4 | 1735646 | low pH vs. bacteria-conditioned | 0.5 | 0.01498501 | Something about silencing protein 4 domain | 3921 |
| 4 | 1735646 | normal vs. bacteria-conditioned | 0.166667 | 0.07592408 | Something about silencing protein 4 domain | 3921 |
| 4 | 1834583 | normal vs. bacteria-conditioned | 0.25 | 0.07092907 | Multicopper oxidase, type 3 | 4200 |
| 4 | 1968854 | normal vs. bacteria-conditioned | 0.333333 | NA | Aldo/keto reductase | 696 |
| 4 | 1968854 | normal vs. low pH | 0.5 | NA | Aldo/keto reductase | 696 |
| 4 | 2305144 | normal vs. low pH | 0.25 | 0.07192807 | FAS1 domain superfamily | 4783 |
| 5 | 38969 | normal vs. bacteria-conditioned | 0.333333 | NA | Longin-like domain superfamily | 3979 |
| 5 | 48790 | low pH vs. bacteria-conditioned | 0.25 | 0.07292707 | RNA recognition motif domain | 1920 |
| 5 | 236388 | low pH vs. bacteria-conditioned | 0.333333 | NA | Ran binding domain | 1329 |
| 5 | 355522 | low pH vs. bacteria-conditioned | 0.333333 | NA | NA | 3831 |
| 5 | 355522 | normal vs. low pH | 0.5 | NA | NA | 3831 |
| 5 | 678520 | normal vs. bacteria-conditioned | 0.25 | 0.06793207 | Fructose-bisphosphate aldolase, class-II | 3860 |
| 5 | 1184523 | low pH vs. bacteria-conditioned | 0.25 | 0.07392607 | Fatty acid desaturase domain | 4206 |
| 5 | 1232754 | low pH vs. bacteria-conditioned | 0.333333 | NA | Vicinal oxygen chelate (VOC) domain | 1376 |
| 5 | 1232754 | normal vs. low pH | 0.333333 | NA | Vicinal oxygen chelate (VOC) domain | 1376 |
| 6 | 639407 | low pH vs. bacteria-conditioned | 0.333333 | NA | Calcineurin-like phosphoesterase domain, ApaH type | 1782 |
| 6 | 639407 | normal vs. bacteria-conditioned | 0.333333 | NA | Calcineurin-like phosphoesterase domain, ApaH type | 1782 |
| 6 | 728208 | normal vs. bacteria-conditioned | 0.5 | NA | Carbohydrate kinase PfkB | 4911 |
| 6 | 728220 | normal vs. bacteria-conditioned | 0.333333 | NA | Carbohydrate kinase PfkB | 4923 |
| 7 | 206974 | normal vs. low pH | 0.25 | 0.08891109 | LicD family | 2246 |
| 8 | 15759 | low pH vs. bacteria-conditioned | 0.722222 | NA | Cytochrome c-like domain | 459 |
| 8 | 15759 | normal vs. bacteria-conditioned | 0.333333 | NA | Cytochrome c-like domain | 459 |
| 9 | 3961 | low pH vs. bacteria-conditioned | 0.333333 | NA | Chaperone DnaK | 2032 |
| 9 | 3961 | normal vs. bacteria-conditioned | 0.5 | NA | Chaperone DnaK | 2032 |
| 9 | 27928 | normal vs. bacteria-conditioned | 0.333333 | NA | Rho protein GDP-dissociation inhibitor | 1130 |
| 10 | 36322 | low pH vs. bacteria-conditioned | 0.333333 | 0.01598402 | DASH complex subunit Ask1 | 2195 |
| 10 | 36322 | normal vs. bacteria-conditioned | 0.5 | 0.00599401 | DASH complex subunit Ask1 | 2195 |
| 50 | 14804 | normal vs. bacteria-conditioned | 0.333333 | NA | Exocyst complex component EXOC3/Sec6, C-terminal domain | 4348 |
| 50 | 14804 | normal vs. low pH | 0.333333 | NA | Exocyst complex component EXOC3/Sec6, C-terminal domain | 4348 |
| 50 | 16264 | normal vs. bacteria-conditioned | 0.25 | 0.0999001 | NA | 4606 |
| 50 | 16264 | normal vs. low pH | 0.25 | 0.0999001 | NA | 4606 |
| 117 | 65833 | low pH vs. bacteria-conditioned | 0.25 | 0.02897103 | NA | 4754 |
| 117 | 65833 | normal vs. low pH | 0.25 | 0.02897103 | NA | 4754 |
| 117 | 1316972 | normal vs. bacteria-conditioned | 0.25 | 0.08991009 | Rho GTPase-activating protein domain | 4144 |
| 117 | 1443605 | low pH vs. bacteria-conditioned | 0.333333 | NA | 2-isopropylmalate synthase | 3828 |
| 117 | 1476712 | normal vs. low pH | 0.333333 | 0.06893107 | TCP-1-like chaperonin intermediate domain superfamily | 3740 |
| 117 | 1481377 | low pH vs. bacteria-conditioned | 0.333333 | NA | Protein kinase domain | 4037 |
| 117 | 1481377 | normal vs. bacteria-conditioned | 0.333333 | NA | Protein kinase domain | 4037 |
| 117 | 1981533 | normal vs. bacteria-conditioned | 0.25 | 0.06993007 | Thioredoxin domain | 3792 |
| 117 | 1984793 | normal vs. bacteria-conditioned | 0.5 | NA | Eukaryotic translation initiation factor 3 subunit J | 2334 |
| 117 | 2030654 | normal vs. bacteria-conditioned | 0.25 | 0.08791209 | Leucine-rich repeat domain superfamily | 4296 |
| 117 | 2034922 | normal vs. bacteria-conditioned | 0.333333 | NA | Mss4 | 4890 |
| 117 | 2097637 | low pH vs. bacteria-conditioned | 0.333333 | NA | Cysteine-rich transmembrane CYSTM domain | 3186 |
| 117 | 2097637 | normal vs. low pH | 0.333333 | NA | Cysteine-rich transmembrane CYSTM domain | 3186 |
| 117 | 2517317 | normal vs. bacteria-conditioned | 0.5 | NA | Protein kinase domain | 4074 |
| 117 | 3462569 | low pH vs. bacteria-conditioned | 0.333333 | NA | Histidine phosphatase superfamily, clade-1 | 4722 |
