# Supplementary material for: Wide-ranging consequences of priority effects governed by an overarching factor
Source: eLife. 2022 Oct 27;11:e79647. doi: 10.7554/eLife.79647 (PMC9671501; doi:10.7554/eLife.79647)
Supplement: Figure 9—source data 3. — Nearest annotated gene to each putative de novo singleton mutation. [file elife-79647-fig9-data3.docx]

### Figure 9-source data 3 - Nearest annotated gene with punitive *de novo* singleton mutation

Nearest annotated gene to each putative *de novo* singleton mutation.

| **Scaffold** | **SNP** | **Treatment** | **Gene ID** | **Signature Description** | **InterPro Description** | **Loci distance to gene (bp)** |
| --- | --- | --- | --- | --- | --- | --- |
| 2 | 142172 | bacteria conditioned | 122804 | SHNi-TPR | Tetratricopeptide, SHNi-TPR domain | 4330 |
| 2 | 152222 | low pH | 122810 | Zn(2)-C6 fungal-type DNA-binding domain profile. | Zn(2)-C6 fungal-type DNA-binding domain | 4580 |
| 2 | 581610 | low pH | 123038 | - | Mitochondrial import inner membrane translocase subunit Tim21 | 3315 |
| 2 | 874406 | normal | 158686 | SET domain profile. | SET domain | 3525 |
| 2 | 983739 | bacteria conditioned | 143523 | Beta-lactamase | Beta-lactamase-related | 3091 |
| 2 | 1193730 | bacteria conditioned | 123348 | SEC7 domain profile. | Sec7 domain | 3703 |
| 2 | 1214463 | bacteria conditioned | 104900 | RNA-binding domain, RBD | RNA-binding domain superfamily | 4296 |
| 2 | 1387782 | low pH | 158874 | Taurine catabolism dioxygenase TauD, TfdA family | TauD/TfdA-like domain | 4020 |
| 2 | 1801552 | ancestral | 143718 | - | Exocyst complex component EXOC6/Sec15, C-terminal, domain 2 | 3974 |
| 2 | 2270572 | low pH | 165272 | ABC transporter integral membrane type-1 fused domain profile. | ABC transporter type 1, transmembrane domain | 4315 |
| 2 | 2613284 | bacteria conditioned | 140298 | HCP-like | NA | 3499 |
| 2 | 2654116 | bacteria conditioned | 124080 | CYTOCHROME C OXIDASE SUBUNIT 6A, MITOCHONDRIAL | NA | 2528 |
| 2 | 2654116 | bacteria conditioned | 124080 | CYTOCHROME C OXIDASE POLYPEPTIDE VIA | Cytochrome c oxidase, subunit VIa | 2528 |
| 2 | 2945111 | bacteria conditioned | 159455 | ER MEMBRANE PROTEIN COMPLEX SUBUNIT 6 | NA | 2215 |
| 2 | 2945111 | bacteria conditioned | 159455 | UNCHARACTERIZED | ER membrane protein complex subunit 6 | 2215 |
| 3 | 15656 | bacteria conditioned | 125316 | - | Tubulin/FtsZ, GTPase domain superfamily | 4228 |
| 3 | 387084 | bacteria conditioned | 125523 | Ferredoxin reductase-type FAD binding domain profile. | FAD-binding domain, ferredoxin reductase-type | 4644 |
| 3 | 792251 | low pH | 140953 | Classic Zinc Finger | NA | 4652 |
| 3 | 1477772 | low pH | 141117 | Protein kinase domain profile. | Protein kinase domain | 3437 |
| 3 | 1535525 | normal | 156507 | Nnf1 | Nuclear MIS12/MIND complex subunit PMF1/Nnf1 | 3517 |
| 3 | 2075720 | low pH | 149825 | ALG6, ALG8 glycosyltransferase family | Glycosyl transferase, ALG6/ALG8 | 3878 |
| 3 | 2144184 | normal | 156735 | Cytidine and deoxycytidylate deaminases domain profile. | Cytidine and deoxycytidylate deaminase domain | 3764 |
| 3 | 2353536 | low pH | 117782 | ARM repeat | Armadillo-type fold | 2219 |
| 3 | 2436246 | normal | 117810 | NNP-1 PROTEIN NOVEL NUCLEAR PROTEIN 1 NOP52 | Nucleolar, Nop52 | 4021 |
| 3 | 2671944 | bacteria conditioned | 117893 | Aldehyde Dehydrogenase; Chain A | Aldehyde dehydrogenase, N-terminal | 4924 |
| 3 | 2671944 | bacteria conditioned | 117893 | Aldehyde Dehydrogenase; Chain A | Aldehyde dehydrogenase, C-terminal | 4924 |
| 3 | 2753650 | low pH | 156963 | Fe(2+) 2-oxoglutarate dioxygenase domain profile. | Oxoglutarate/iron-dependent dioxygenase | 4708 |
| 3 | 2909079 | bacteria conditioned | 117984 | Solute carrier (Solcar) repeat profile. | Mitochondrial substrate/solute carrier | 4378 |
| 3 | 3082088 | normal | 118051 | TTL domain profile. | Tubulin-tyrosine ligase/Tubulin polyglutamylase | 4200 |
| 4 | 79542 | normal | 118841 | t-SNARE coiled-coil homology domain profile. | Target SNARE coiled-coil homology domain | 2595 |
| 4 | 262947 | normal | 141816 | Regulator of G-protein signaling, RGS | RGS domain superfamily | 4830 |
| 4 | 286255 | normal | 112888 | Prokaryotic membrane lipoprotein lipid attachment site profile. | NA | 1486 |
| 4 | 414723 | bacteria conditioned | 138051 | Eukaryotic RNA Recognition Motif (RRM) profile. | RNA recognition motif domain | 2087 |
| 4 | 756357 | low pH | 138127 | - | Fe-S cluster assembly domain superfamily | 2038 |
| 4 | 1536342 | low pH | 160157 | Acetyl-coenzyme A (CoA) carboxyltransferase N-terminal domain profile. | Acetyl-coenzyme A carboxyltransferase, N-terminal | 3642 |
| 4 | 1692875 | low pH | 113406 | PRA1 family protein | Prenylated rab acceptor PRA1 | 325 |
| 4 | 1780889 | bacteria conditioned | 160246 | Forkhead-associated (FHA) domain profile. | Forkhead-associated (FHA) domain | 3677 |
| 4 | 1966106 | normal | 142199 | AKR | Aldo/keto reductase | 3444 |
| 4 | 2163139 | low pH | 154278 | Pyoverdine/dityrosine biosynthesis protein | Pyoverdine/dityrosine biosynthesis protein | 636 |
| 4 | 2190640 | bacteria conditioned | 119880 | Ubiquitin specific protease (USP) domain profile. | Ubiquitin specific protease domain | 3341 |
| 5 | 38989 | normal | 124511 | SNARE-like | Longin-like domain superfamily | 3959 |
| 5 | 39790 | normal | 124511 | SNARE-like | Longin-like domain superfamily | 3158 |
| 5 | 252114 | low pH | 154487 | Candida agglutinin-like (ALS) | Agglutinin-like protein repeat | 3285 |
| 5 | 252114 | low pH | 154487 | Candida agglutinin-like (ALS) | Agglutinin-like protein repeat | 3285 |
| 5 | 252114 | low pH | 154487 | Candida agglutinin-like (ALS) | Agglutinin-like protein repeat | 3285 |
| 5 | 549641 | bacteria conditioned | 116652 | Translation proteins SH3-like domain | Translation protein SH3-like domain superfamily | 2612 |
| 6 | 319227 | normal | 161928 | MFS general substrate transporter like domains | NA | 3992 |
| 6 | 319531 | ancestral | 161928 | MFS general substrate transporter like domains | NA | 3878 |
| 6 | 427980 | bacteria conditioned | 118319 | TLDc domain profile. | NA | 1247 |
| 6 | 528907 | normal | 141612 | PLA2c domain profile. | Lysophospholipase, catalytic domain | 3786 |
| 6 | 686853 | low pH | 141643 | TLC domain profile. | TRAM/LAG1/CLN8 homology domain | 3970 |
| 6 | 699796 | normal | 118472 | Major facilitator superfamily (MFS) profile. | Major facilitator superfamily domain | 1840 |
| 6 | 980857 | bacteria conditioned | 118638 | alpha/beta-Hydrolases | Alpha/Beta hydrolase fold | 862 |
| 6 | 992775 | normal | 118648 | SIGMA 1-TYPE OPIOID RECEPTOR-RELATED | ERG2/sigma1 receptor-like | 4387 |
| 6 | 1127358 | low pH | 118711 | Mu homology domain (MHD) profile. | Mu homology domain | 4349 |
| 8 | 2881 | normal | 157102 | - | NA | 3261 |
| 8 | 64919 | low pH | 103781 | TB2/DP1, HVA22 family | TB2/DP1/HVA22-related protein | 3451 |
| 9 | 26756 | bacteria conditioned | 125192 | RHO protein GDP dissociation inhibitor | Rho protein GDP-dissociation inhibitor | 2302 |
| 54 | 7419 | normal | 120011 | Serine proteases, subtilase domain profile. | NA | 4015 |
| 54 | 11135 | low pH | 120011 | Serine proteases, subtilase domain profile. | NA | 299 |
| 54 | 11152 | bacteria conditioned | 120011 | Serine proteases, subtilase domain profile. | NA | 282 |
| 54 | 11862 | low pH | 120011 | Serine proteases, subtilase domain profile. | NA | 737 |
| 54 | 12493 | normal | 120011 | Serine proteases, subtilase domain profile. | NA | 1368 |
| 117 | 20485 | bacteria conditioned | 160460 | ARM repeat | Armadillo-type fold | 974 |
| 117 | 20595 | bacteria conditioned | 160460 | ARM repeat | Armadillo-type fold | 864 |
| 117 | 341822 | bacteria conditioned | 120229 | Transcription mediator complex subunit Med12 | Mediator complex, subunit Med12 | 3644 |
| 117 | 592420 | bacteria conditioned | 120359 | Zinc finger C2H2 type domain profile. | Zinc finger C2H2-type | 4695 |
| 117 | 668209 | bacteria conditioned | 120406 | Glycosyl hydrolases family 18 | Glycoside hydrolase family 18, catalytic domain | 3687 |
| 117 | 925961 | bacteria conditioned | 163279 | Nucleotide-diphospho-sugar transferases | Nucleotide-diphospho-sugar transferases | 4190 |
| 117 | 1330373 | low pH | 163422 | Protein kinase domain profile. | Protein kinase domain | 4774 |
| 117 | 1330453 | low pH | 163422 | Protein kinase domain profile. | Protein kinase domain | 4854 |
| 117 | 1331269 | bacteria conditioned | 120799 | Allantoicase repeat | Allantoicase domain | 3260 |
| 117 | 1392788 | normal | 142560 | Zn(2)-C6 fungal-type DNA-binding domain profile. | Zn(2)-C6 fungal-type DNA-binding domain | 4298 |
| 117 | 1550134 | normal | 120918 | Transmembrane amino acid transporter protein | Amino acid transporter, transmembrane domain | 3766 |
| 117 | 1578280 | low pH | 120929 | - | NA | 1510 |
| 117 | 1685479 | bacteria conditioned | 163532 | USP_Like | NA | 2067 |
| 117 | 1685494 | bacteria conditioned | 163532 | USP_Like | NA | 2052 |
| 117 | 1685560 | normal | 163532 | USP_Like | NA | 1986 |
| 117 | 1698523 | low pH | 163538 | UNCHARACTERIZED | Pre-rRNA-processing protein Esf1 | 4656 |
| 117 | 1850710 | ancestral | 121089 | - | NA | 1113 |
| 117 | 1914706 | bacteria conditioned | 121132 | SAM-dependent MTase RsmB/NOP-type domain profile. | SAM-dependent methyltransferase RsmB/NOP2-type | 4575 |
| 117 | 2011919 | normal | 114390 | - | NA | 3312 |
| 117 | 2322449 | low pH | 121409 | Cyclin_C_H_G | NA | 2345 |
| 117 | 2375781 | low pH | 161383 | - | HAD superfamily | 3555 |
| 117 | 2393784 | bacteria conditioned | 161388 | WD40 repeat-like | WD40-repeat-containing domain superfamily | 1282 |
| 117 | 2395046 | normal | 121448 | Histone-fold | Histone-fold | 4332 |
| 117 | 2499913 | low pH | 163813 | Universal stress protein signature | Universal stress protein A family | 2742 |
| 117 | 2499913 | low pH | 163813 | Universal stress protein signature | Universal stress protein A family | 2742 |
| 117 | 2499913 | low pH | 163813 | Universal stress protein signature | Universal stress protein A family | 2742 |
| 117 | 2681072 | bacteria conditioned | 161494 | Protein kinase domain profile. | Protein kinase domain | 1015 |
| 117 | 2925974 | low pH | 163965 | Heavy-metal-associated domain profile. | Heavy metal-associated domain, HMA | 2173 |
| 117 | 3039368 | normal | 147163 | Vps54-like protein | Vacuolar protein sorting-associated protein 54, C-terminal | 4292 |
| 117 | 3198640 | low pH | 103079 | - | WD40/YVTN repeat-like-containing domain superfamily | 2 |
