# Supplementary material for: Wide-ranging consequences of priority effects governed by an overarching factor
Source: eLife. 2022 Oct 27;11:e79647. doi: 10.7554/eLife.79647 (PMC9671501; doi:10.7554/eLife.79647)
Supplement: Supplementary file 1. — Translation of the English abstract in some of the other languages spoken by the authors. [file elife-79647-supp1.docx]

**Abstract (German)**

Prioritätseffekte treten in Ökosystemen auf, wenn die Reihenfolge des Eintreffens verschiedener lokaler Arten und deren anfängliche relative Häufigkeit die Interaktionen der Arten untereinander beeinflussen. So können Prioritätseffekte den Zustand eines Ökosystems beeinflussen, was wiederrum taxonomische, funktionale und evolutionäre Einflüsse auf die ökologische Artengemeinschaft haben kann. Es ist noch unklar, ob die weitreichenden Folgen von Prioritätseffekten systematisch durch einen gemeinsamen zugrunde liegenden Faktor erklärt werden können. Wir haben einen solchen Faktor in einem empirischen System identifiziert. In einer Reihe von Feld- und Laborstudien haben wir erforscht, wie sich der pH-Wert auf nektarbesiedelnde Mikroben und deren Interaktionen mit Pflanzen und Bestäubern auswirkt. In einer Feldstudie fanden wir heraus, dass die mikrobiellen Gemeinschaften im Nektar in einem von Kolibris bestäubten Strauch, *Diplacus* (bisher: *Mimulus*) *aurantiacus*, Häufigkeitsmuster aufweisen, die auf alternative stabile Zustände hinweisen, die durch die Dominanz von Bakterien oder Hefen in einzelnen Blüten entstehen. Darüber hinaus variierte der pH-Wert des Nektars zwischen den Blüten von *D. aurantiacus*, was weiterhin auf die Existenz dieser alternativen stabilen Zustände hindeutet. In Laborexperimenten übte *Acinetobacter nectaris*, das am häufigsten im Nektar von *D. aurantiacus* vorkommende Bakterium, einen stark negativen Prioritätseffekt gegenüber *Metschnikowia reukaufii*, der am häufigsten vorkommenden und auf Nektar spezialisierten Hefe, aus, indem es den pH-Wert des Nektars senkte. Dieser Prioritätseffekt erklärt wahrscheinlich das sich gegenseitig ausschließende Dominanzmuster zwischen den beiden Arten, das bei der Felduntersuchung festgestellt wurde. Darüber hinaus führten wir ein Evolutionsexperiment durch, in dem wir die Ausbreitung der Mikroben zwischen Blüten simuliert haben, die normalerweise durch Kolibris stattfindet. Das Experiment zeigte, dass *M. reukaufii* sich schnell weiterentwickeln kann, um die Resistenz gegenüber des Prioritätseffektes zu verbessern, solange es dauerhaft einer Reduktion des pH-Wertes ausgesetzt ist, die durch *A. nectaris* hervorgerufen wird. Schließlich fanden wir in einem Feldexperiment heraus, dass ein niedriger pH-Wert des Nektars den Nektarverbrauch durch Kolibris verringern kann, was auf funktionelle Folgen des pH-gesteuerten Prioritätseffekts für die Pflanzenreproduktion hindeutet. Zusammengenommen zeigen diese Ergebnisse, dass es möglich ist, einen übergreifenden Faktor (in unserem Fall der pH-Wert) zu identifizieren, der die öko-evolutionäre Dynamik von Prioritätseffekten auf mehreren Ebenen der biologischen Organisation eines Ökosystems bestimmt.

**Abstract (Hindi)**

प्राथमिकता प्रभाव, जहां आगमन क्रम और प्रारंभिक प्रचुरता स्थानीय प्रजातियों की परस्पर क्रिया को नियंत्रित करते हैं, वे पारिस्थितिक समुदायों पर टैक्सोनोमिक, कार्यात्मक और विकासवादी प्रभाव डाल सकते हैं। यह स्पष्ट नहीं है कि प्राथमिकता के प्रभावों के इन व्यापक परिणामों को एक सामान्य आधारभूत कारक द्वारा व्यवस्थित रूप से समझाया जा सकता है। यहां, हम एक अनुभवजन्य प्रणाली में ऐसे कारक की पहचान करते हैं। प्रकृति और प्रयोगशाला अध्ययनों की एक श्रृंखला में, हम इस बात पर ध्यान केंद्रित करते हैं कि पी.एच. कैसे अमृत-उपनिवेशीकरण रोगाणुओं और पौधों और परागणकों के साथ उनकी परस्पर क्रिया को प्रभावित करता है। एक क्षेत्र सर्वेक्षण में, हमने एक हमिंगबर्ड (चिड़िया)-परागणित झाड़ी, डिप्लैकस ऑरेंटियाकस में अमृत सूक्ष्मजीव समुदायों में या खमीर या बैक्टीरिया का फूलों में हावी होना पाया। इसके अलावा, अमृत पी.एच. इन प्रभुत्व प्रवृत्ति के अनुसार फूलों के बीच भिन्न होता है।

प्रयोगशाला प्रयोगों में, एसिनेटोबैक्टर नेक्टारिस, डी. ऑरेंटियाकस अमृत में सबसे अधिक पाया जाने वाला जीवाणु, अमृत पी.एच. को कम करके, सबसे आम अमृत-विशेषज्ञ खमीर, मेट्सनिकोविया रेउकॉफी के खिलाफ एक जोरदार नकारात्मक प्राथमिकता प्रभाव डाला। यह प्राथमिकता प्रभाव संभवतः क्षेत्र सर्वेक्षण में पाए गए प्रभुत्व के पारस्परिक रूप से अनन्य प्रवृत्तियों की व्याख्या करता है। इसके अलावा, फूलों के बीच हमिंगबर्ड-सहायता प्राप्त फैलाव का अनुकरण करने वाले प्रायोगिक विकास से पता चला कि एम। रेउकॉफी प्राथमिकता प्रभाव के खिलाफ प्रतिरोध में सुधार करने के लिए तेजी से क्रमागत विकास कर सकता है। अंत में, एक क्षेत्र प्रयोग में, हमने पाया कि कम अमृत पी.एच. हमिंगबर्ड द्वारा अमृत का उपभोग करना कम कर सकता है, जो पौधे के प्रजनन के लिए पी.एच.-संचालित प्राथमिकता प्रभाव का सुझाव देता है। एक साथ लिया गया, इन परिणामों से पता चलता है कि एक व्यापक कारक की पहचान करना संभव है, जो जैविक संगठन के कई स्तरों पर प्राथमिकता प्रभावों के पर्यावरण-विकासवादी गतिशीलता को नियंत्रित करता है।

**Abstract (Japanese)**

種の移入順序や初期密度が違うと異なる種間相互作用が生じることがあり、この現象は先住効果とよばれています。先住効果は生物群集の代替安定状態を作り出し、群集の種構成や生態系の機能、種の形質進化などにさまざまな変化が起きることがあります。しかしこれら多岐にわたる影響は共通の根本的な要因によって体系的に説明できるかどうかは不明のままです。ここでは、そのような要因の発見について報告します。この研究は米国カリフォルニア州にみられる低木*Diplacus* (または*Mimulus*) *aurantiacus*、この低木の主な送粉者であるハチドリ、そしてこの低木の花の蜜に棲む微生物の関係を対象にしています。まず、野外観察によって、個々の花の蜜では細菌または酵母のいずれかが優占し、細菌と酵母の両者が同じ花に多く見られることはほとんどないことが分かりました。また、細菌が優占すると蜜のpH値が極度に低まり、酵母が優占すると中程度に低まることが分かりました。これらの結果は細菌の優占と酵母の優占が代替安定状態となっている可能性を示唆しています。さらに、室内実験によって、*D. aurantiacus*の蜜に最もよく見られる細菌*Acinetobacter nectaris*は、蜜のpH を下げることで、*D. aurantiacus*の蜜に最もよく見られる酵母*Metschnikowia reukaufii*に強い負の先住効果を及ぼすことが確かめられました。この先住効果が、野外観察でみられた細菌と酵母の相互に排他的な分布様式を引き起こしていると考えられます。また、別の室内実験では、蜜のpHが細菌によって常に低い値をとる状況では、先住効果に対する耐性を強めるような急速な進化が酵母に起きることも明らかになりました。さらに、野外実験によって、蜜のpHが低いとハチドリによる蜜の消費が減ることが分かりました。以上の結果をまとめると、先住効果の影響が多岐にわたる場合でも、全てひとつの要因で包括的に説明できることが示されました。

**Abstract (Mandarin)**

物種到達的先後順序和初始相對豐度能夠調節當地物種的相互作用，此過程稱為先驅者效應。先驅者效應可以推動生態群落到不同的替代狀態，從而對其物種組成、功能和演化產生影響。目前研究還沒能找到一個單一成因系統性地解釋先驅者效應。此研究通過一個實驗系統發現一個如此的因素。透過一系列的野外調查和實驗，我們探討了酸鹼（pH）值如何影響花蜜微生物以及它們與植物和傳粉者的相互作用。在一項野外調查中，我們發現在蜂鳥授粉的一種灌木植物 (Diplacus aurantiacus) 中，花蜜微生物群落表現出先驅者效應；單個花朵內的細菌和酵母菌表現出替代穩定狀態。此外，D. aurantiacus 花蜜的pH值隨著相應的替代穩定狀態變化。在實驗中，D. aurantiacus 花蜜中最常見的細菌Acinetobacter nectaris降低花蜜的pH值，從而對最常見的花蜜酵母Metschnikowia reukaufii產生強烈的負面效應。這種先驅者效應解釋了野外調查所發現的相互排斥的物種主導狀態。此外，在模擬蜂鳥花粉傳播的演化實驗中，酵母菌M. reukaufii可以快速演化來適應A. nectaris導致的pH值降低，從而抵消先驅者效應。最後，在一野外實驗中，我們發現低花蜜pH值可以降低蜂鳥對花蜜的消耗，這表明pH值驅動的先驅者效應對植物繁殖有功能性的影響。綜上所述，我們透過野外調查及實驗發現了支配先驅者效應的生態演化的首要因素。

**Abstract (Spanish)**

Los efectos de prioridad, cuando el orden de llegada y la frecuencia inicial modulan interacciones locales entre especies, pueden ejercer influencias taxonómicas, funcionales y evolutivas sobre las comunidades ecológicas, produciendo estados estables alternativos. Una posibilidad es que estas consecuencias diversas podrían ser explicadas sistemáticamente por un solo factor subyacente. En este trabajo, identificamos empíricamente uno de estos factores. Conjuntando estudios de campo y de laboratorio, nos enfocamos en cómo el pH del néctar floral afecta a microbios que colonizan el propio néctar, y a sus interacciones con plantas y polinizadores. En un estudio de campo, encontramos que las comunidades microbianas en el arbusto *Diplacus* (anteriormente *Mimulus*) *aurantiacus*, el cual es polinizado por colibríes, muestran distribuciones de abundancia indicativas de estados estables alternativos, caracterizados respectivamente por la dominancia de bacterias o levaduras en flores individuales. Adicionalmente, el pH del néctar de *D. aurantiacus* muestra una distribución consistente con la existencia de estos estados estables alternativos. En experimentos de laboratorio, *Acinetobacter nectaris*, la bacteria más comúnmente identificada en el néctar de *D. aurantiacus*, reduce el pH del néctar y ejerce un efecto de prioridad fuertemente negativo sobre *Metschnikowia reukaufii*, la especie más común de levadura especialista en néctar. Este efecto de prioridad probablemente explica el patrón de dominancia y exclusión mutua encontrado en el estudio de campo. Además, en un estudio de evolución experimental, en el que simulamos dispersión entre flores por colibríes, encontramos que *M. reukaufii* puede evolucionar rápidamente para incrementar su resistencia contra el efecto de prioridad cuando está expuesto a la reducción de pH inducida por *A. nectaris*. Finalmente, en otro estudio de campo, encontramos que el néctar con pH bajo es menos consumido por colibríes, sugiriendo que el efecto de prioridad causado por el pH puede tener consecuencias funcionales para la reproducción de la planta. En conjunto, estos resultados muestran que se puede identificar un solo factor, que gobierna la dinámica eco-evolutiva de los efectos de prioridad en múltiples niveles de organización biológica.
